# Tumors Responsive to Autophagy-Inhibition: Identification and Biomarkers

**DOI:** 10.3390/cancers12092463

**Published:** 2020-08-31

**Authors:** Lydie M.O. Barbeau, Tom G.H. Keulers, Kasper M.A. Rouschop

**Affiliations:** Department of Radiation Oncology (Maastro), GROW School for Oncology and Developmental Biology, Maastricht University Medical Centre+, 6200MD Maastricht, The Netherlands; l.barbeau@maastrichtuniversity.nl (L.M.O.B.); tom.keulers@maastrichtuniversity.nl (T.G.H.K.)

**Keywords:** autophagy-addiction, biomarkers, personalized medicine, cancer

## Abstract

**Simple Summary:**

Although the principle of personalized medicine has been the focus of attention, many cancer therapies are still based on a one-size-fits-all approach. The same holds true for targeting cancer cell survival mechanism that allows cancer cells to recycle their constituents (autophagy). In the past several indicators of elevated dependence of cancer cells on autophagy have been described. Addition of autophagy-inhibiting agents could be beneficial in treatment of these tumors. The biomarkers and mechanisms that lead to elevated dependence on autophagy are reviewed in the current manuscript.

**Abstract:**

Recent advances in cancer treatment modalities reveal the limitations of the prevalent “one-size-fits-all” therapies and emphasize the necessity to develop personalized approaches. In this perspective, identification of predictive biomarkers and intrinsic vulnerabilities are an important advancement for further therapeutic strategies. Autophagy is an important lysosomal degradation and recycling pathway that provides energy and macromolecular precursors to maintain cellular homeostasis. Although all cells require autophagy, several genetic and/or cellular changes elevate the dependence of cancer cells on autophagy for their survival and indicates that autophagy inhibition in these tumors could provide a favorable addition to current therapies. In this context, we review the current literature on tumor (sub)types with elevated dependence on autophagy for their survival and highlight an exploitable vulnerability. We provide an inventory of microenvironmental factors, genetic alterations and therapies that may be exploited with autophagy-targeted approaches to improve efficacy of conventional anti-tumor therapies.

## 1. Introduction

Despite improvements in cancer therapy, cancers frequently relapse, illustrating the limitations of the current “one-size-fits-all” therapies. In part, this is related to the limited characterization of each individual tumor with its own genetic and phenotypic characteristics. Comprehensive analysis and identification of the tumor’s vulnerability will help to move forward into designed therapies with targeted treatments that are more effective in individual patients. 

Genetic and microenvironmental inter- and intra-tumor differences in cancers result in biological reprogramming that increase reliance on specific signaling pathways for progression and survival. Identification of biomarkers that indicate pathway-addiction will aid in selecting the most effective treatment regimen between tumors of the same origin. This includes targeting of the “self-eating” mechanism, macroautophagy (hereafter, autophagy), which is frequently induced during cellular stress including hypoxia, nutrient deprivation and chemotherapy [1].

Autophagy is a homeostatic cellular catabolic process in which damaged and old cytoplasmic components (e.g., proteins and organelles) are sequestered into a double-membrane vesicle to be further degraded through lysosomal fusion. The products of lysosomal breakdown (e.g., amino acids and fatty acids) are recycled and transported across the lysosomal membrane into the cytoplasm. These metabolites can be reused in protein and ATP production [2]. During metabolic stress or periods of insufficient nutrient supply, autophagy can be rapidly upregulated to support energy production and provide building blocks for essential cellular functions.

For example, directly after birth, neonatal mice are exposed to nutrient starvation as the trans-placental nutrient supply has ceased. Autophagy enables survival to neonatal starvation through prevention of energy depletion until it is restored by milk intake [3]. In line, cells deprived of glucose or amino acids, or exposed to hypoxia, depend on autophagy for their survival [4,5,6]. In short, autophagy acts as a pro-survival mechanism through maintaining energy homeostasis and removal of toxic protein aggregates, pathogens and old/damaged organelles [7]. During conditions of starvation or metabolic stress, autophagy plays an important role by “recycling” amino acids and nutrients to maintain energy levels, protein synthesis and essential metabolic processes [8]. Furthermore, autophagy is essential for mitochondrial removal that might otherwise become cytotoxic [8,9,10,11,12]. 

In the context of cancer, (contextual) synthetic lethality is classically defined as the setting in which (in)activation of individual genes, nutrient status or microenvironment has limited effect on cell viability. The dependence on the supportive or compensating gene or mechanism results an attractive target in cancer therapy, mostly via pharmacological inhibition [13,14]. In contrast to normal cells, some cancer cells display elevated basal autophagy activity [15,16] and depend on autophagy for their survival [17,18]. Autophagy is highly responsive to nutrient availability and energy status [17,18], suggesting that tumors highly dependent on specific nutrients or have insufficient supply will be more responsive to autophagy-targeting therapies [19]. In the past years, several cellular alterations have been identified that increase the dependence of specific cancer cells on autophagy and may be important as biomarker in predicting the efficacy of autophagy inhibition in cancer therapy. 

Combined targeting of autophagy with other targeted drugs could open a new window for further therapeutic strategies. However, the evidence that autophagy inhibition is an effective complementary strategy in cancer therapy is limited [20,21,22,23]. This may be related to lack of use of predictive biomarkers that identify autophagy-dependent tumors in patient populations and stresses the use of these biomarkers for future clinical trials. 

In the past, selection of patient groups was frequently based on identifying increased autophagy activity, but this is not necessarily associated with elevated dependence on autophagy for survival. In addition, the role of autophagy (pro- or anti-survival) in cancer may be context dependent [24,25], highlighting the necessity to determine which tumor types and genetic/environmental/therapy backgrounds can synergize with autophagy targeting and are useful for patient stratification [26]. 

Here, we review cancer genotypes and metabolic statuses that increase cellular dependence on autophagy for survival. These include cancer cells with metabolic alterations (hypoxia and glutamine addiction), drug exposure (tyrosine kinase inhibitors), alterations due to changes in oncogenic signaling (e.g., MAPK, UPR and mTOR) or mutations (e.g., EGFRvIII and RAS). In addition, we list several emerging biomarkers that are associated with improved response to autophagy-inhibitory strategies (Table A1). These mechanisms and potential biomarkers illustrate a context dependent role for autophagy and advocate the principles of personalized medicine in trial design that include autophagy targeting.

## 2. Head and Neck Cancer 

### 2.1. Hypoxia

The majority of solid human tumors, including glioblastoma, contain regions that are poorly oxygenated (hypoxia), a very heterogeneous and dynamic feature. Hypoxia is associated with a more malignant phenotype due to higher resistance to chemo- and radiotherapy and increased metastasis development. From a clinical point of view, reducing tumor hypoxia is highly desired since low oxygenation of tumors is associated with poor outcome in multiple cancer types [27]. Reduced oxygenation induces autophagy [6,28]. Although tumor hypoxia is a common feature of solid tumors and contributes to therapy resistance and disease progression in many cancer types, the evidence is strongest in head and neck cancer (HNSCC) [29]. In HNSCC, a meta-analysis indicated that the degree of hypoxia is the most significant factor explaining variability in survival [30] and demonstrated therapeutic benefit of hypoxia modification [31]. In multiple cancers, including HNSCC, expression of the autophagy related proteins microtubule associated protein 1 light chain 3 beta (LC3B), autophagy-related 5 (ATG5), unc-51-like autophagy activating kinase 1 (ULK1), BNIP and BCL2 interacting protein-like 3 (BNIP3L) is induced during hypoxia, indicating the importance for autophagy activation for survival [6,32,33]. Failure to induce these genes leads to autophagy inhibition and hypoxic cell death. In line, pharmacological targeting of autophagy results in elevated exposure to reactive oxygen species (ROS), a reduction in tumor hypoxia and sensitization of tumors to therapy, indicating that HNSCC tumors with high hypoxia will respond to autophagy inhibition [6,10,34]. Hypoxia associated patient stratification should therefore be considered before combining conventional therapies with autophagy-targeting. 

### 2.2. PTOV1 Expression

Independent of hypoxia, recent studies have suggested that prostate tumor-overexpressed gene 1 (PTOV1) could serve as prognostic biomarker in nasopharyngeal and laryngeal tumors [35,36]. Expression of PTOV1 induces autophagy and is associated with increased resistance to cisplatin in vitro and in laryngeal cancer patients [37]. Targeting autophagy (hydroxychloroquine) in patient-derived cell lines that were cisplatin resistant (high PTOV1) resulted in vast cell killing, indicating the autophagy dependence and suggests that PTOV1 could be used as biomarker for selection of laryngeal cancer patients that would likely benefit from concurrent autophagy targeting. 

## 3. Brain Cancer

### 3.1. EGFRvIII Expression

Glioblastoma (GBM) is the most malignant form of glioma and autophagy activation results in resistance to temozolomide [23,38], suggesting that autophagy targeting in combination with conventional therapy may have beneficial effects on therapy. A significant population of glioblastoma expresses a truncated EGFR receptor, epidermal growth factor receptor variant III (EGFRvIII), which is associated with increased hypoxia tolerance, radio- and chemoresistance and pro-survival [39]. Recently, we showed that ectopic and endogenous expression of EGFRvIII, in support of the high metabolic demand, increases dependence on autophagy for survival during hypoxia [34]. Adding the autophagy inhibitor chloroquine (CQ) reduces the viable hypoxic fraction and, subsequently, radiosensitized EGFRvIII GBM tumors. A previous study by Sotelo et al. indicated in a placebo controlled trial the clinical benefit of adding CQ to the treatment of surgery, carmustine and radiotherapy, extending patient survival to 24 months compared to 11 months for the control arm [40]. Retrospective analyses including patients of the same cohort indicated that patients with EGFRvIII^+^ tumors displayed the largest benefit. The incidence of EGFRvIII^+^ tumors is highly abundant (53%) in this cohort and may have contributed to the positive effect in the overall cohort [34]. Although not designed to test efficacy, patient stratification in a recently conducted phase 1 trial showed a similar trend that treatment of EGFRvIII^+^ glioblastoma benefit from combined treatment with CQ, temozolomide and radiotherapy [41]. 

A clinical trial conducted by Rosenfeld et al. did not yield a survival advantage in glioblastoma patients after targeting autophagy with hydroxychloroquine (HCQ) in combination with radiotherapy and temozolomide. Dose-limiting toxicity prevented escalation to higher doses of HCQ, and autophagy inhibition was not consistently achieved in patients treated with this regimen. In addition, the representation of EGFRvIII^+^ tumors has not been assessed [42]. Additionally, most drugs (including CQ and HCQ) are lysosomotropic drugs that accumulate in lysosomes due to the low PH. In general, the tumor microenvironment is acidic and may protonate autophagy inhibitors and prevent their diffusion over cellular membranes and limit their effectiveness [43]. 

One of the key regulators of autophagy activity is mammalian target of rapamycin (mTOR). Activation of mTOR supports cell proliferation and inhibits autophagy [44,45]. EGFR signaling stimulates activation of mTOR, resulting in an inverse relation of EGFR-expression to autophagy activity in cells. The residual autophagy is essential for cellular survival as the level of EGFR expression is associated with elevated cell killing after autophagy inhibition. 

### 3.2. BRAF^v600E^

In addition to mutations and amplification of EGFR, abnormal activation of RAS/RAF/MEK/ERK pathway frequently occurs in cancer due to mutations in other membrane receptors, downstream effectors such as KRAS and BRAF or alternative pathways that regulate RAF activity such as phosphoinositide 3-kinase (PI3K), phosphatase and tensin homolog (PTEN) and protein kinase B (AKT) [46]. In high-grade pediatric central nervous system (CNS) tumors, *BRAF^V600E^* mutations are frequently observed (10–30% incidence) [47]. The expression of *BRAF^V600E^* drives autophagy activation and autophagy dependence for survival [48]. Combination of a *BRAF^V600E^* specific inhibitor, vemurafenib, with CQ synergistically improved outcome for a young patient diagnosed with a second recurrence of ganglioglioma [48]. The *BRAF^V600E^* mutation could be used as a selective marker to implement autophagy inhibitors in the treatment of childhood brain tumors [49]. In addition, despite an initial response, BRAF-driven cancer cells can become resistant to *BRAF^V600E^* kinase inhibition. Using early or late autophagy inhibitors (i.e., SBI-0206965, VPS34 knockdown, CQ, *ATG5* knockdown and *ATG7* knockdown) could overcome acquired resistance to BRAF inhibitors in brain tumor patients [50,51], indicating an important role for autophagy inhibition to prevent drug resistance.

### 3.3. Neurotrophins

Deregulation of growth factor receptors and aberrant secretion of their ligands contributes to malignancy of glioma [52]. Neurotrophins (NTs) and tropomyosin-related kinase receptor (Trk) are important in the development of the nervous system [53], but also contribute to tumor progression [54]. Jawhari et al. observed high expression of TrkC and NT-3 in sections of glioblastoma [55]. During periods of hypoxia, p38MAPK-induced TrkC/NT-3 signaling and autophagy are required to maintain mitochondrial homeostasis and promote glioblastoma cell survival. Expression of LC3B is most pronounced in hypoxic areas of the tumor [6]. In glioblastoma, high TrkC and NT-3 were associated with hypoxia and colocalized with LC3B and BNIP3. Targeting autophagy increased TrkC/NT-3 signaling and reduced proliferation, suggesting interdependence of these pathways. Combining CQ with TrkC-targeting resulted in vast cell killing, particularly in hypoxic cells [55]. Targeting autophagy in glioblastoma with elevated TrkC/NT-3 expression and/or signaling could be considered in future clinical trials.

### 3.4. WNT-Signaling

Many studies have suggested that WNT signaling is aberrantly activated in glioblastoma and that it promotes to glioblastoma growth and invasion via the maintenance of stem cell properties (reviewed in [56]). A recent study suggests that GBM harboring low WNT-CTNNB1 signaling or CTNNB1 mutations could benefit of autophagy inhibition [57]. CTNNB1/β-catenin is the principal effector of WNT canonical pathway and controls the expression of WNT target genes together with the transcription factor of the TCF/LEF families. In glioblastoma, hCTNNB1 mutations are rare and WNT signaling activation occurs mainly through upregulation of WNT ligands or epigenetic regulation of WNT inhibitors. Glioblastoma cell proliferation is maintained through canonical and non-canonical WNT signaling by the WNT pathway component disheveled segment polarity protein 2 (DLV2). Autophagy inhibits WNT signaling by promoting DLV2 and CTNNB1 degradation. In contrast, the TCF–CTNNB1 complex represses autophagy by increasing AKT/MAPK/mTOR signaling and by inactivating the transcription factor EB (TFEB), a master regulator of lysosomal biogenesis and sequestosome 1 (SQSTM1) expression. Inhibition of WNT signaling through targeting TCF4 and autophagy flux reduces tumor growth, through a SQSTM1-dependent mechanism and caspase 8 activation. Combined targeting of WNT signaling and autophagy may therefore be beneficial in glioblastoma with high WNT signaling, but may also suggest that tumors with low WNT-CTNNB1 with high autophagy-SQSTM1 expression will be sensitive to autophagy inhibition [57].

In summary, high EGFR, EGFRvIII^+^ and *BRAF^V600E^* mutated glioblastoma and/or childhood tumors present a dependency on autophagy signaling. Autophagy targeting could be considered to improve outcome after therapy. In addition, TrkC and WNT-CTNNB1 expression status might identify potential glioblastoma susceptible to autophagy inhibitors in combination with targeting agents.

## 4. Pancreatic Cancer

Autophagy inhibition (HCQ) combined with gemcitabine/nab-paclitaxel is relatively well tolerated by patients, as demonstrated in preoperative phase 1–2 trials. Combination treatment improves pathological response, serum CA 19-9 levels and lymph node involvement and increases immune cell infiltration. However, the combined treatment does not improve overall and progression-free survival, indicating that autophagy could be an important therapeutic target in patients with pancreatic ductal adenocarcinoma (PDAC) but requires further optimization for optimal use in clinical contexts (NCT01128296 and NCT01978184 [58,59,60,61]). Several molecular markers and mechanisms in PDAC that have been linked to autophagy-addiction may be considered. 

### 4.1. KRAS

PDAC are characterized with a high rate of *KRAS* mutations (approximately 95%), reprogrammed metabolism, hypervascular and hypoxic microenvironment and an invasive nature. Autophagy plays a critical role in *KRAS*-driven PDAC development and progression in response to hypoxia, nutrient deprivation and chemotherapy [62,63]. PDAC depend on autophagy to promote tumor proliferation and suppress cell death [16,64], support their nutritional requirements [65,66] and preserve metabolic homeostasis [15].

In late stage disease, *KRAS* mutation is often accompanied by a *p53* loss of heterozygosity and high autophagy activity that dictates the progression of the pancreatic cancer. Mice developing *Kras^G12D/+^*, *p53^+/−^, Atg5^−/−^* PDAC display better overall survival. Blocking autophagy significantly compromises cell survival and patient-derived xenograft (PDX) growth independently of *p53* mutational load [67]. Interestingly, autophagy impairment in the early stage of development (when *p53* is still intact) may prevent the transformation of neoplastic pancreatic cancer towards invasive PDAC [68,69]. However, *p53* has a dual role in regulating autophagy based on its cellular localization. In the nucleus, *p53* activates autophagy through mTOR inhibition and AMP-activated protein kinase (AMPK) pathway, while cytoplasmic *p53* inhibits autophagy, indicating that *p53* status and localization has to be considered before the addition of autophagy inhibitors [70,71].

In addition, phospholipase D1 (PLD1) mediates autophagy [72] and modulates AMPK and mTOR (in) activation [73]. Suppression of AMPK results in increased PLD1 activity, resulting in increased phosphatidic acid (PA) production. Conversely, PLD1 inhibition increases ULK1 phosphorylation [73], and PLD1 knockdown results in inhibition of starvation induced autophagy [72], suggesting that PLD1 status may be used as biomarker to identify tumors that would benefit of targeting autophagy.

Nonetheless, other reports indicate that autophagy is dispensable to promote *KRAS*-driven cell growth [74,75], and autophagy addiction seems to be context-dependent. Cell type specific effects dictate whether oncogenic *HRAS* or *KRAS* mutations will stimulate or inhibit CQ-mediated toxicity [76]. This illustrates that oncogenic RAS determination is insufficient as a single biomarker to predict autophagy-inhibition efficacy and should only be considered in combination with additional biomarkers. 

### 4.2. EI24 Expression

*EI24* (etoposide-inducible autophagy associated transmembrane) is a *p53*-inducible gene that is frequently lost in breast and cervical cancers (17–41%). EI24 suppresses growth and functions as an effector of *p53*-mediated tumor progression. EI24 is an essential autophagy gene for execution of basal autophagy and clearance of aggregate-prone proteins [77]. Although the initial loss of EI24 and the associated loss of autophagy activity may have contributed to tumorigenesis [78], loss of EI24 at later stages impairs proliferation and cell survival in pancreatic cancer cells. In vivo, autophagy reduction through EI24 deficiency is not sufficient to significantly decrease tumor growth [79]. Although it is tempting to speculate that EI24-deficient tumors are unlikely to respond to autophagy inhibition, on several occasions we observed that autophagy targeting did not influence tumor growth, but was capable of inducing alterations in the tumor microenvironment that influence responsiveness to therapy (e.g., hypoxia) [6,34]. In addition, comparable to EGFR overexpressing glioblastoma, low autophagy activity, as observed in EI24 ^low^ cells, could increase the dependence of PDAC on residual autophagy. The potential of EI24 expression as biomarker for targeting autophagy in PDAC requires further investigation.

### 4.3. MAPK/ERK Inhibitors

Clinical use of MAPK/ERK inhibitors has shown some beneficial effects in the treatment of PDAC. Unfortunately, innate or acquired resistance is frequently observed and is a major limitation in effective use. In parallel, single autophagy inhibition in patients indicates limited efficacy and fails to improve outcome (NCT04132505 [80,81]). Noteworthy, in vitro data show promising response with the combination MAPK/ERK and autophagy inhibitors [82]. In *KRAS* WT or mutant tumors, pharmacological inhibition of ERK (SCH772984) or *KRAS* (ARS-1620) induces autophagy directly via increasing autophagy related gene expression and indirectly by altering cell metabolism (glycolysis) [83]. Autophagy inhibition synergizes with ERKi and enhances ERKi-mediated growth suppression in *KRAS*-mutant PDAC. 

Notably, synergistic effects of KRAS/RAF/MEK/ERK inhibitors combined with autophagy inhibitors is not restricted to *KRAS*-driven PDAC models, but is also observed in *NRAS*-mutated melanoma, *BRAF*-mutated colorectal patient derived xenografts (PDXs) models [84] and *BRAF*-mutant thyroid cancers [85]. Kinsey et al. showed that patients benefit from the combination of MEK-inhibition (trametinib) with CQ after relapsing disease after first line treatment [84]. Additional clinical trials are currently determining the recommended dose of HCQ in combination with trametinib for future phase 2 trials in *RAS* mutant PDAC (NCT03825289 [86]).

### 4.4. Glutamine Addicted Cancer

Pancreatic cancer cells rewire intermediary metabolism to support different energetic and biosynthetic demands [87]. Alterations in nutrient uptake and catabolism, such as aberrant consumption of the nonessential amino acid glutamine, drive anabolic growth under hypoxic conditions and after oncogenic pathway dysregulation [88,89,90]. In absence of glucose, glutamine is the second main source of carbon to fuel the tricarboxylic acid cycle (TCA) cycle and the primary source of nitrogen for nucleotide and amino acid synthesis [79]. Through glutaminolysis, glutamine provides availability of nicotinamide adenine dinucleotide phosphate (NADPH), required for lipid synthesis and maintenance of the cellular redox balance. Insufficient delivery of glucose therefore increases the dependence on glutamine metabolism under lack of oxygen [91]. Glutamine limitation activates autophagy and in KRAS-driven PDAC, autophagy compensates the lack of glutamine via the degradation of intracellular macromolecules [92]. Conversely, blockage of autophagy lowers intracellular glutamine levels leading to cell apoptosis [19]. In line, we observed that HNSCC cancer cells that receive insufficient oxygen and glucose rapidly elevated autophagy activity. This provides the cell with liberation glutamine, as illustrated by the increased availability of non-protein associated extra- and intracellular glutamine during hypoxia (data not shown). The inability to active autophagy rapidly depletes intracellular glutamine [19], resulting in increased tumor cell killing and improved patient prognosis in several cancer types. Amino acids/glutamine transporters, solute carrier family 3 member 2 (*SLC3A2*/CD98hc) and solute carrier family 7 member 5 (*SLC7A5*/LAT1), expression could serve as markers of autophagy dependence. After chemo- and radiotherapy, absence of *SLC3A2* impaired amino acid exchange and activation of mTOR/PI3K pathway to allow autophagy related gene expression. *SLC3A2*-deficient tumor cells rely on autophagy during glutamine deficiency and are radiosensitized after autophagy inhibition [93]. However, autophagy impairment during glutamine deprivation can reprogram the metabolic and transcriptional cell landscape, notably regulators of the TCA cycle [94] and may lead to increased expression of amino acids transporters (AATs) to compensate lack of glutamine and generate energy [18,95]. Blocking autophagy in combination with glutaminolysis inhibitor (e.g., C.968) has been shown to be an effective strategy in glutamine-addicted tumors [96]. 

In conclusion, inhibiting autophagy could synergize with current standard of care in *KRAS* mutant PDAC at early stage of development (p53 present), at late stage with p53 loss of heterozygosity and high autophagy activity or PDAC with low EI24 activity to enhance injurious effect. This therapy could be extendable to autophagy addicted RAS-driven cancers [24] and glutamine-addicted (*SLC3A2*-deficient) PDAC and HNSCC tumors.

## 5. Melanoma

### BRAF^v600E^

The *BRAF^V600E^* mutation is the most common *BRAF* alteration (80%) and an important oncogenic driver in melanomas, pediatric CNS, lung cancer (3%) and colorectal cancer (6–12%) [97,98]. Melanomas present the highest alteration rate with over 90% of *BRAF*^V600E^ mutations, accompanied with 30–40% mutations in *PTEN*. Autophagy is essential for survival of cells with *BRAF^V600E^* mutations [48,99,100], maintenance of lung cancer cell metabolic homeostasis [101,102] and development of treatment resistance in colorectal cancer cells [103].

In genetically engineered mouse models (GEMMs) that yield *Braf^V600E^*- and Pten-deficient melanomas, tumor-specific ablation of *Atg7* resulted in accumulation of defective mitochondria and impaired tumor growth. Pharmacological targeting of *BRAF^V600E^* (dabrafenib) enhances the anti-proliferative effect [104], indicating that autophagy inhibition in combination with *BRAF^V600E^* inhibitors could provide an effective strategy in treatment of melanomas. The treatment of melanomas *BRAF^V600E^* inhibitor has proven to be an effective therapy. Nevertheless, melanoma frequently relapses due to acquired resistance. Ma et al. identified that melanomas with highest resistance to *BRAF^V600E^* inhibitors displayed elevated levels of autophagy. In vitro inhibition of *BRAF^V600E^* resulted in activation of the unfolded proteins response due to sequestration of *BRAF^V600E^* in the endoplasmic reticulum and resulted in activation of cytoprotective autophagy. Inhibition of autophagy in combination with *BRAF^V600E^* targeting resulted in tumor regression [105]. Considering the encouraging results, phase 1 and 2 clinical trials have been initiated that combine *BRAF^V600E^* inhibitors or MEK inhibitors (downstream of BRAF-signaling) with HCQ (NCT01897116 [106] and NCT02257424 [107]).

## 6. Colorectal Cancer

### 6.1. KRAS

*KRAS* mutations account for approximately 30–50% of oncogenic driving mutations in colorectal cancer (CRC). Similar to *BRAF^V600E^*, *KRAS* alterations induce autophagy through MEK/ERK signaling and contribute to CRC cell survival [108]. Comparable to observations in pancreatic cancer, genetic ablation of autophagy in *KRAS*-driven CRC tumors can either promote or inhibit tumor growth driven by activation of different signaling pathways. Regressing autophagy-deficient tumors display an induction of the NOTCH signaling and a reduction in mitochondrial activity. Tumors that grow despite autophagy ablation rely on PI3K/AKT/mTOR signaling and a decrease in glucose metabolism for their expansion [109]. 

Signal transduction of *KRAS*-mutant CRC cell line is dependent on effective RAF networks. Lee et al. identified BRAF, CRAF and ATG7 as the best target combination to minimize toxicity in WT *KRAS* cells and normal tissue effects but maximize the effect in the *KRAS*-mutated cells. Co-targeting autophagy and MAPK pathways, by silencing *ATG7* together with therapeutic agents against MEK, BRAF and CRAF (trametinib and RAF709), spared normal cells, while inducing apoptosis in KRAS mutated CRC and also PDAC cells [110].

### 6.2. JNK-Signaling

Similar to other MAPKs, the c-Jun N-terminal kinase (JNK) proteins are activated by a series of phosphorylation events in response to multiple stimuli such as cytokines, growth factors, pathogens, stress, toxins and drugs to promote tumorigenesis. During hypoxia, the PKCδ (protein kinase Cδ)/JNK1 axis promotes autophagy through Bcl-2 phosphorylation followed by dissociation from Beclin1 [111]. JNK1 inhibition impaired hypoxia-induced autophagy and resulted in increased cell death [112]. The validity of JNK1 as potential biomarker for autophagy-dependence is currently being evaluated in a phase 1/2 clinical trial in CRC (NCT01206530 [113]).

### 6.3. TFEB

Lysosomal interaction is an essential part of autophagy. TFEB regulates transcription of lysosomal genes and can be trailed through its translocation from the cytoplasm to the nucleus [114]. In non-small cell lung cancer (NSCLC), TFEB presence is associated with high levels of lysosomal markers and a poor prognosis [115]. In CRC, nuclear TFEB drives doxorubicin-induced autophagy and prevents drug-associated apoptosis. Genetic or pharmacological inhibition of autophagy partially reverses drug-resistance and results in increased cell death [116]. 

In short, driver mutations in *BRAFV ^600E^* or *KRAS*, JNK1 expression and nuclear localization of TFEB are indicative of CRC tumors that are susceptible to autophagy targeting and clinical evaluation should be considered. 

## 7. Breast Cancer

### 7.1. JAK/STAT Signaling

Autophagy is important in mammary tumorigenesis [117,118] and alterations in important autophagy-regulatory effectors such as PI3K, p53, EGFR and Bcl-2 proteins; PI3K/mTOR signaling [119]; and Janus kinases–signal transducer and activator of transcription proteins (JAK/STAT) pathways are frequently observed [120]. Constitutive activation of STAT3 signaling is observed in 50–60% of breast cancer events. This pathway is key in transducing extracellular signals (cytokines, interferons or growth factors) into transcriptional programs that regulate cell growth, invasiveness, metastasis and maintenance of cancer stem cell (CSC) viability [120,121]. Autophagy inhibition particularly affects stem cells, cytokine secretion and epithelial-to-mesenchymal transition (EMT) in breast cancer cells. Lacking a leader sequence [122], autophagy controls IL6 secretion and is required for CSC viability [123]. Interestingly, elevated production of interleukin 6 (IL6) correlates with increased *p*-STAT3 and contributes to the paracrine activation of signal transducer and activator of transcription 3 (STAT3) [124]. Breast cancer cells highly active in STAT3 activity display increased dependency on autophagy for their survival. Advantageously, combining autophagy inhibitors with doxorubicin synergistically inhibits tumor growth in triple-negative breast cancer (TNBC) with high JAK/STAT pathway activation, advocating for autophagy inhibition in this subtype of tumors [125].

### 7.2. HER2 Signaling

HER2 (human epidermal growth factor receptor 2) is a transmembrane receptor tyrosine kinase (RTK) of the EGFR family and HER2 inhibition is a common strategy for breast cancers with HER2 amplification. Transphosphorylation of the protein dimer stimulates downstream signaling, including MAPK cascades, PI3K/AKT/mTOR pathway or STAT transcription factors, all known regulators of autophagy [126]. Administration of RTK inhibitors induces autophagy, which supports the development of resistance mechanisms [127,128]. After acquisition therapy resistance, HER2+ tumors maintain a higher basal level of autophagy to support their proliferation [129,130]. Recent preclinical studies indicate that autophagy inhibition with HER2-targeted inhibitors may enhance tumor cell death in breast cancer cells as autophagy impairment restores sensitivity to HER2 inhibitors in breast cancer and in lapatinib-resistant EAC cell line (*HER2*-amplified esophageal adenocarcinoma) [131,132,133]. In line, monoallelic loss of *BECN1* is frequent in HER2+ breast cancers and predicts response to HER2-inhibitors (trastuzumab) [134]. *BECN1* overexpression enhances HER2 phosphorylation/activation and decreases response to RTK inhibitors (lapatinib). Conversely, Beclin1 knockdown improved lapatinib-induced apoptosis [135], indicating that HER2 treatment efficacy is largely determined by the autophagy-status of the cell. Targeting HER2 signaling in combination with autophagy inhibitors is an appealing strategy. 

Phase 1 and 2 clinical trials are currently ongoing to evaluate the asset of autophagy inhibitors alone [136,137,138] or together with other chemotherapeutic agents, such as microtubules inhibitors (taxane and taxane-like drugs) [139], mTOR inhibitor (everolimus) [140] and PI3K/mTOR inhibitors (gedatolisib) [141]) in breast cancer treatment.

### 7.3. Estrogen Receptor Signaling

Estrogen receptor-positive (ER+) breast cancer is effectively treated with endocrine therapies. In response to endocrine therapy, autophagy is induced and contributes to resistance to anti-hormone therapies [142,143,144]. In line, tamoxifen-resistant breast cancer cells display high autophagy activation combined with overexpression of metastasis-associated 1 (MTA1). In ER+ breast cancer patients treated with tamoxifen, MTA1 expression increases in relapsed or recurrent disease and correlates with low disease free survival. Knockdown of MTA1 decreases autophagic activity, indicating a direct role for MTA1 in regulation of autophagy. Importantly, pharmacological or genetic inhibition of autophagy restores tamoxifen sensitivity, indicating that autophagy inhibitory strategies could be considered for tamoxifen resistant, high MTA1 expressing breast cancer [145].

### 7.4. CDK4 and-6 Inhibition

Alternatively, ER+/HER2− breast cancers may be treated with cyclin-dependent kinase CDK4 and CDK6 inhibitors (e.g., palbociclib, abemaciclib or ribociclib). CDK4/CDK6/cyclin D signaling is altered in 35% of ER+ breast cancer patients, but 16% of patients fail to respond to the treatment. CDK4/CDK6 inhibition increases autophagy. Interestingly, the inhibition of CDK4/CDK6 in combination with genetic (siRNA *Beclin1* or siRNA *ATG5*) or pharmacological (HCQ, spautin-1 or bafilomycin A1) autophagy targeting induces a synergistic anti-proliferative effect in vitro and diminishes growth of patient-derived xenografts isolated from retinoblastoma-associated protein positive (Rb+) and low-molecular-weight isoforms of cyclin E (LMWE) cytoplasmic negative (LMWE cyt−) breast cancer [146]. In summary, LMWE appears to be predictive in sensitivity for the combination of CDK4/CDK6 inhibitors with autophagy targeting in advanced ER+ breast cancer [146].

### 7.5. IKBKE and JMJD6

Both inhibitor of nuclear factor kappa B kinase subunit epsilon (IKBKE) and Jumonji domain-containing 6 (JMJD6) contribute to enhancement of tumor supportive autophagy. In a myristoylated screen, IKBKE was identified as an inducer of autophagy in triple negative and in HER2-positive breast cancers. Active IKBKE-induced autophagy supports oncogenic cellular transformation of immortalized mammary cells and is required for TNBC cell proliferation, suggesting that targeting autophagy represents a feasible approach to prevent TNBC evolution and progression [147].

JMJD6 overexpression results in increased malignancy, such as increased migration and poor differentiation that eventually leads to poor prognosis. A newly discovered intrinsic tyrosine kinase domain in JMJD6 is required for H2AX ^Y39^ phosphorylation and mediates transcriptional regulation of ATG genes in TNBC. Impairment of kinase activity and autophagy suppresses TNBC growth in vivo, implying that JMJD6 could serve as predictive biomarker for autophagy inhibition in breast cancer [148].

In summary, targeting autophagy appears to be a profitable approach in several breast cancer subtypes. In HER2+, autophagy inhibition reverses acquired tyrosine kinase inhibitors (TKIs) resistance and enhances cell mortality. IKBKE activation identifies HER2+ as well as TNBC tumors susceptible to autophagy inhibition. In TNBC, treatment opportunities remain limited, although could benefit from autophagy inhibition when displaying high STAT3 activity or high JMJD6 expression. Finally, MTA1 may identify receptive tamoxifen-ER+ resistant cells and their dependence to autophagy pathway.

## 8. Lung Cancer

### 8.1. TKI

In NSCLC, activating mutations in *EGFR* are favorable predictors for EGFR TKIs-based therapies. Although EGFR TKIs can prolong survival, different acquired resistance mechanisms have been identified. Although the degree of induction varies according to the drug and the cancer cell lines [149,150], autophagy activation is systematic and recurrent upon TKIs independently of the target and the cancer type. Cytoprotective autophagy has been observed in mutant and wild-type *EGFR* NSCLC [150,151,152] and in *EGFR* mutant bladder cancer [153]. Inhibiting autophagy-mediated TKI-resistance may be considered as therapeutic strategy to overcome resistance TKIs in EGFR deregulated cancers [21,82,154,155]. Considering the positive pre-clinical data, addition of autophagy inhibitor (HCQ) to standard of care erlotinib is currently being evaluated in patients with mutant or high expressing EGFR NSCLC tumors (NCT00977470 [156]).

### 8.2. KRAS and LKB1 Signaling

Similar to PDAC, autophagy plays an important role in KRAS- and p53-driven lung cancer tumorigenesis and metabolism [157,158]. In Western countries, *KRAS* and liver kinase B1 (*LKB1* or *STK11*) are the most common oncogene driver mutations in NSCLC (~40% of cases) [159,160]. Tumor suppressor gene *LKB1* encodes an upstream kinase of AMPK, which enables the dissociation between ULK1 and mTOR thereby allowing ULK1 association with ATG13/FIP200 to activate autophagy [44,161]. Loss of LKB1 reprograms lung cancer metabolism to generate energy and sustain cell proliferation. Upon starvation, LKB1 mutant human lung cancer cells depend on autophagy to maintain energy homeostasis by recycling substrates for TCA metabolism and biogenesis of fatty acids. Moreover, autophagy is required for tumor initiation and progression and energy homeostasis in Lkb1-deficient Kras-driven NSCLC GEMMs. Atg7 and Lkb1 loss sensitize tumor-derived cell lines (TDCLs) to metabolic stress, suggesting that autophagy inhibition can be a favorable approach for the treatment of Lkb1-deficient lung tumors [162,163].

In summary, acquired resistance to TKIs in EGFR wild-type and mutant tumors (e.g., HER2+ breast, bladder and lung) is (partially) reversed by autophagy inhibitors. Lkb1 deficiency identifies tumors that are highly vulnerable to autophagy-inhibition due to their sole dependence on autophagy to meet its metabolic demand. 

## 9. Liver Cancer

### HGF Signaling

Hepatocyte growth factor (HGF) and MET signaling supports cellular proliferation, promotes epithelial-mesenchymal transition (EMT) and causes invasion and metastasis during malignant transformation in hepatocellular carcinoma (HCC). MET kinase activity plays a key role in cancer cell metabolism through induction of glycolysis, glutamine synthesis through increased glutaminase activity, glutaminolysis and lipid synthesis [164]. Interestingly, MET contains two distinct LC3-interacting regions (LIRs) close to two tyrosine phosphorylation sites and orchestrates the autophagic response. Dephosphorylated/inactive MET interacts with LC3 and promotes autophagy activity. In 51% of HCC patients, dephosphorylated MET associated with absence of SQSTM1 was observed, suggesting SQSTM1 degradation through high autophagy activity. Combined targeting of MET-activation and autophagy synergistically reduces cell viability and results in HCC regression. These results suggest that TKI treatment of HCC with high HGF-MET signaling activity and autophagy-targeting should be considered for effective therapy [164].

Autophagy is essential for CD24-mediated resistance to sorafenib. CD24 is glycoprotein that is highly expressed in tumors including HCC and mediates kinase inhibitor resistance through PP2A/AKT/mTOR inactivation and activation of lysosomal degradation. Blocking autophagy with bafilomycin A1 or knocking-down *ATG5* overcomes sorafenib resistance in CD24 high expressing HCC, suggesting that CD24 could be used as a biomarker for therapeutic benefit for autophagy-inhibition in sorafenib-resistant HCC [165].

## 10. Prostate Cancer

### KDMB4 Expression

Pten and Atg7-autophagy loss in GEMMs revealed tumor-promoting effects of autophagy in castrate-naïve and castrate-resistant prostate cancers (PCa) [166]. The histone lysine demethylase KDM4B fulfills a critical role in this signaling by co-activating androgen and estrogen receptors [167,168]. In PCa, KDM4B stimulates androgen receptor (AR) transcription activity via histone demethylation and AR ubiquitination [169]. High KDM4B expression correlates with resistance to androgen deprivation treatment in PCa patients [170]. In castration-resistant prostate cancer (CRPC) cells, KDM4B expression stimulates autophagy activation through Wnt/beta-catenin signaling and sequestration of beta-catenin in the nucleus. Autophagy inhibition reverses the growth advantage of KDM4B expressing cells, suggesting that high KDM4B expression identifies CRPC patients that could benefit from autophagy targeted therapy [170].

## 11. Bone Cancer

### HSP90AA1

Bone cancers are rare at less than 1% of all cancers. Osteosarcoma and chondrosarcoma are the two main subtypes of primary bone malignancies. Chemotherapy is generally not very effective for chondrosarcoma, while it is an important part of the treatment for osteosarcoma. Therapy resistance can arise through expression of conserved chaperone heat shock proteins (HSPs). HSP90AA1-induced doxorubicin-resistance inhibits cell death through deactivation of JNK and p38 kinase signals and stimulates pro-survival autophagy through PI3K/AKT/mTOR signaling inhibition. Hence, targeting HSP90AA1 or autophagy recovery restores sensitivity to chemotherapy induced killing. These results suggest that HSP90AA1^high^ osteosarcoma could benefit from concurrent autophagy inhibition [171].

## 12. Conclusions

As observed with other new discoveries and therapeutic intervention possibilities in oncology (e.g., immunotherapy, apoptosis and extracellular vesicles), the “one size fits all” approach does not apply to autophagy inhibition as well. Although there are some common mechanisms or microenvironmental stresses that increase the dependence on autophagy for survival, intrinsic differences determine the efficacy of autophagy inhibitory strategies. Identification of biomarkers that predict autophagy-addiction in combination with autophagy inhibition appears to be the most logical and most effective strategy. 

**Figure 1:**
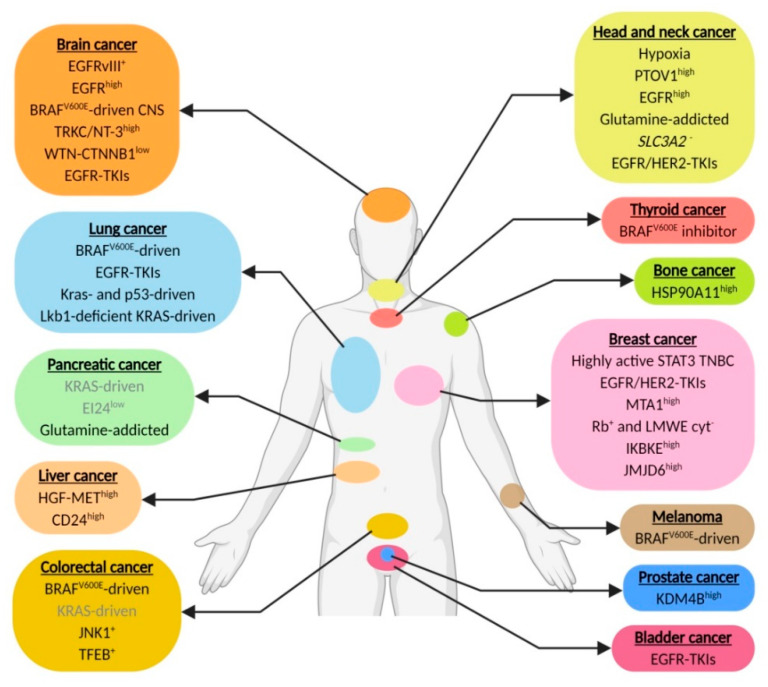
Genetic changes and chemotherapy-resistance mechanisms that contribute to elevate autophagy dependence. Biomarkers in grey require further investigation (created using Https://Biorender.com).

**Figure 2:**
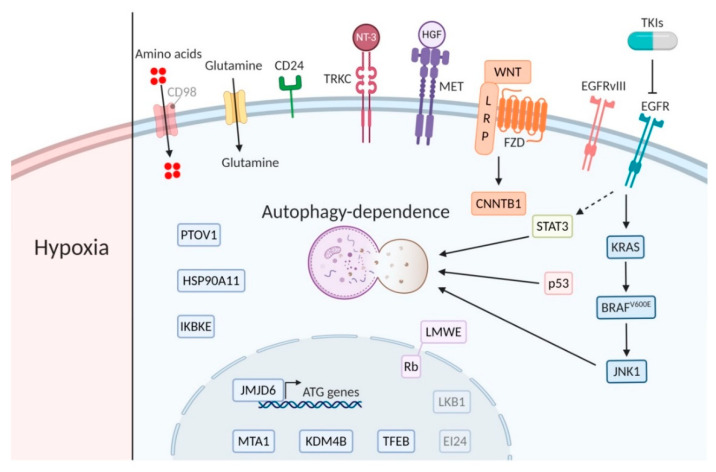
Major players of the main signaling pathways. Bright biomarkers indicate amplification or active signaling and blurred biomarkers indicate low activity or expression in relation to autophagy dependence (created using Https://Biorender.com).

In this context, we review the current literature and list the different cancer (sub)types that pre-clinically or in more advanced stages have been associated with increased dependence on autophagy for survival. These include genetic changes, e.g., EGFR, *EGFRvIII* and *BRAF^v600E^*, that increase dependence on autophagy to supply in the high metabolic demand and chemotherapy-resistance mechanisms, e.g., against TKI and chemotherapy (Figure 1 and Figure 2). The assessment of autophagy dependence is however complex and is often assumed to reveal itself by increased autophagy activity. Nevertheless, an important point is that high autophagy flux does not guarantee an addiction to the signaling and can be seen as a “side effect” of other pathway alterations without contributing to cells survival. In addition, low autophagy activity in some cases dictates sensitivity to autophagy inhibitors [172], most likely due to high dependence on the residual autophagy activity to maintain cellular homeostasis. Unfortunately, in most cancer (sub)types where reduced autophagy activity is observed, no assessment of autophagy-dependence will be done.

Clinical trials in different cancer types have been conducted and demonstrated the relative safety of targeting autophagy with CQ or HCQ. Nevertheless, several trials have not been able to provide patients with the maximum intended dose before reaching the maximum tolerated dose [42]. To date, it remains impossible to measure autophagy inhibition in patients at the site where autophagy inhibition is intended, the tumor, without taking sequential biopsies. In most circumstances, this is not possible or undesired due to the associated risk and extra burden for the patient. Whether sufficient autophagy inhibition in the tumor is reached to successfully increase therapy efficacy remains a matter of investigation. Without having tools available that indicate whether sufficient autophagy inhibition is reached, the evaluation of the success of autophagy-inhibitory strategies will be dependent on improving outcome for patients after therapy. Patient stratification and selection for tumor types with biomarkers that have shown promising results in preclinical studies will determine the feasibility of autophagy inhibition in oncological care.

## Appendix A

**Table A1:** Tumor (sub)types and biomarkers associated with autophagy-dependence and their improved combination therapies.

Cancer Type	Tumor Type	Biomarker/Factor	Mechanism of Autophagy Dependency (When Known)	References
Head and neck cancer	HNSCC	Hypoxia	Nutrient suppletion, reduction ROS exposure	[6,10,33]
	Laryngeal cancer cisplatin or 5-FU resistant	PTOV1^high^	Cisplatin resistance	[37]
	HNSCC	EGFR^high^	-	
	HNSCC	Glutamine	Glutamine suppletion	NP*
	HNSCC	*SLC3A2* ^-^	-	[93]
	Esophageal HER2 amplification	TKIs against EGFR and HER2	-	[133]
Brain cancer	Hypoxic glioblastoma	EGFRvIII^+^	Nutrient suppletion	[34]
	Glioblastoma	EGFR^high^	-	[172]
	Central nervous system	BRAF^V600E^	-	[47,48]
	Gliomas	TRKC/NT-3^high^	Hypoxia tolerance	[55]
	Glioblastoma	WNT-CTNNB1^low^ or mutated	-	[57]
	Neuroblastoma	TKIs against EGFR or multiple TKIs or Axl	-	
Pancreatic cancer	Kras-driven	Kras	-	[16,64]
			-	[67]
			-	[84]
	PDAC	EI24^low^	-	[79]
	KRAS-driven	Glutamine	Glutamine suppletion	[19]
Thyroid cancer	BRAF^V600E^-driven	BRAF^V600E^ inhibitor	-	[85]
Skin cancer	Melanoma	BRAF^V600E^	Removal of mutated protein aggregates	[104]
Colorectal cancer	CRC	BRAF^V600E^	-	[103]
	KRAS-driven	KRAS	-	[110]
	CRC, metastatic CRC or adenocarcinoma	JNK1^+^	-	[113]
	Doxorubicin resistant	TFEB^+^	-	[116]
Breast cancer	TNBC	STAT3	-	[125]
	HER2+	TKIs against HER2	TKI resistance	[131]
		TKIs against EGFR and HER2	TKI resistance	[132]
	ER+ breast cancer tamoxifen resistant	MTA1^high^	Tamoxifen resistance	[145]
	Breast cancer with Rb^+^ and LMWE cyt^-^ or Letrozole or Anastrozole resistant	Rb^+^ and LMWE cyt^-^	-	[146]
	TNBC and HER2-	IKBKE^high^	-	[147]
	TNBC	JMJD6^high^	JMJD6 phosphorylates H2A.X^Y39^ and regulates ATG genes transcription	[148]
Lung cancer	Braf^V600E^-driven	Braf^V600E^	-	[101]
	NSCLC with *EGFR* WT and mutant	TKIs against EGFR	-	[151]
			-	[152]
	Kras- and p53-driven	Kras	-	[157,158]
	Lkb1-deficient Kras-driven NSCLC	Lkb1 (STK11)	TCA and fatty acid synthesis support	[163]
Bladder cancer	*EGFR* mutant	TKIs against EGFR and HER2	-	[153]
Liver cancer	HCC	HGF-MET^high^	-	[164]
	HCC sorafenib resistant	CD24^high^	Sorafenib resistance	[165]
Prostate cancer	Castration-resistant	KDM4B^high^	-	[170]
Bone cancer	Osteosarcoma doxorubicin or cisplatin or methotrexane resistant	HSP90A11^high^	Doxorubicin resistance	[171]

*NP: non-published observation.
